# High stretch cycling inhibits the morphological and biological decidual process in human endometrial stromal cells

**DOI:** 10.1002/rmb2.12341

**Published:** 2020-07-20

**Authors:** Ryohei Saito, Takeshi Kajihara, Masashi Takamura, Hideno Tochigi, Tsuyoshi Sato, Osamu Ishihara

**Affiliations:** ^1^ Department of Obstetrics and Gynecology Saitama Medical University Iruma‐gun Japan; ^2^ Department of Oral and Maxillofacial Surgery Saitama Medical University Iruma‐gun Japan

**Keywords:** cyclic stretch, endometrial decidualization, forkhead box O1, insulin‐like growth factor‐binding protein 1, prolactin

## Abstract

**Purpose:**

Subendometrial myometrium exerts wave‐like activity throughout the menstrual cycle, and uterine peristalsis is markedly reduced during the implantation phase. We hypothesized that abnormal uterine peristalsis has an adverse effect on the endometrial decidualization process. We conducted an in vitro culture experiment to investigate the effect of cyclic stretch on the morphological and biological endometrial decidual process.

**Methods:**

Primary human endometrial stromal cells (HESCs) were isolated from hysterectomy specimens and incubated with or without 8‐bromo‐cyclic adenosine monophosphate (8‐br‐cAMP) and medroxyprogesterone acetate (MPA) for 3 days. After decidualization, cultures were continued for 24 hours with or without cyclic stretch using a computer‐operated cell tension system.

**Results:**

Cyclic stretch significantly repressed expression of decidual markers including insulin‐like growth factor‐binding protein 1 (*IGFBP1*), prolactin (*PRL*), forkhead box O1 (*FOXO1*), and *WNT4* on decidualized HESCs. In addition, cyclic stretch of decidualized HESCs affected the decidual morphological phenotype to an elongated shape. The alternation of F‐actin localization in decidualized HESCs was not observed in response to cyclic stretch.

**Conclusions:**

These data suggest that cyclic stretch inhibits the morphological and biological decidual process of HESCs. Our findings imply that uterine abnormal contractions during the implantation period impair endometrial decidualization and contribute to infertility.

## Graphical abstract

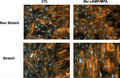

## 1 INTRODUCTION

Successful pregnancy requires coordination of three interdependent processes: embryo development, endometrial differentiation, and placenta formation. Decidualization is a morphological and biological transformation of human endometrial stromal cells (HESCs) into decidual cells, and it is essential for blastocyst implantation and subsequent formation of the placenta.^1^, ^2^ The most characteristic morphological alteration in the decidual process is the drastic transformation of the endometrial stromal fibroblasts into secretory epithelioid decidual cells.^3^ At the molecular level, decidual transformation involves extensive reprogramming of many cell functions mediated by the activation of various key transcription factors. The convergence of these transcription factors on promoters of key genes drives the expression and secretion of major decidual factors including prolactin (*PRL*), *WNT4*, and insulin‐like growth factor‐binding protein 1 (*IGFBP1*).^3^, ^4^ Impairment of the decidualizing process is increasingly linked to a variety of pregnancy disorders including infertility, recurrent implantation failure, recurrent miscarriages, preeclampsia, intrauterine growth restriction, endometriosis, and endometrial malignancy.^5^, ^6^, ^7^, ^8^

It has been reported that subendometrial myometrium exerts wave‐like activity (uterine peristalsis) throughout the menstrual cycle,^9^ and uterine peristalsis is markedly reduced during the implantation phase. The unique feature of uterine peristalsis may facilitate implantation of the embryo to the endometrium. A clinical study reported that none of the enrolled patients with intramural myomas in a high‐frequency peristalsis group achieved pregnancy, whereas one‐third of patients in the low‐frequency peristalsis group became pregnant.^10^ Furthermore, myomectomy reduced the frequency of uterine peristalsis in patients who had exhibited an abnormally high‐frequency of peristalsis prior to surgery. In addition, myomectomy increased the pregnancy rate in patients who had exhibited high‐frequency peristalsis.^11^ These observations suggest that abnormal uterine peristalsis during the implantation period influences endometrial functions and contributes to infertility. Harada et al^12^ found that endometrial peristalsis elevates IGFBP‐1 levels and promotes the decidualization of HESCs. The effect of physiological stretching conditions had been already investigated in that study. However, the effect of abnormal stretching conditions on HESCs is unclear. Therefore, we hypothesized that abnormal uterine peristalsis has an adverse effect on the endometrial decidual process. We conducted an in vitro culture experiment to investigate the effect of cyclic stretch on the morphological and biological endometrial decidualization process.

## 2 MATERIALS AND METHODS

### 2.1 Tissue collection and isolation of HESCs

Human endometrium was collected from patients undergoing hysterectomy for uterine fibroids at Saitama Medical University Hospital. Institutional Review Board approval (17‐085) was obtained for this research project. All women had regular menstrual cycles and were not receiving hormonal treatment at the time of surgery. Informed consent was obtained from all patients before tissue collection. The age of participants was 44.5 ± 4.9. All the endometrial tissues were obtained from women in proliferative phase. HESCs were isolated as previously described.^6^, ^13^, ^14^, ^15^ Harvested HESCs were cultured in maintenance medium of DMEM/F‐12 (Thermo Fisher Scientific, Waltham, MA, USA) containing 10% dextran‐coated charcoal‐treated FBS supplemented with 2 μg/mL insulin from bovine pancreas (Sigma‐Aldrich, St. Louis, MO, USA), 1 × 10^−9^ M beta‐estradiol (Sigma‐Aldrich), 1% antibiotic‐antimycotic solution (Thermo Fisher Scientific), and 1% L‐glutamine solution (Thermo Fisher Scientific) according to the previous study.^6^

### 2.2 Mechanical loading experiment

Confluent monolayers on a BioFlex six‐well plate (Flexcell International Corporation, Hillsborough, NC, USA) were treated with or without 0.5 mmol/L 8‐bromo‐cAMP (8‐br‐cAMP; Sigma‐Aldrich) and 10^−6^ M medroxyprogesterone acetate (MPA; Sigma‐Aldrich) as decidual stimulation for three days. Decidualized or non‐decidualized HESCs were then subjected to cyclic sinusoidal equibiaxial tensile strain (amplitude, 0% to 8%; frequency, 0.1 Hz: 6 cycles/min; sine waveform) for 24 hours using the Flexcell FX‐4000™ Tension System (Flexcell^®^ Tension Plus System; Flexcell Corporation, McKeesport, PA, USA) according to the manufacturer's instructions. Culture medium had not been changed after the start of decidual stimuli. All experiments were performed before the fourth cell passage.

### 2.3 The aspect ratio

Because cell elongation is a morphologic indicator of abnormal cyclic stretch, we determined the aspect ratio of each cell's major axis of cell length over the minor axis of cell width (with an aspect ratio of 1.0 yielding a circle) for the HESCs in culture using ImageJ software. The shape indexes were calculated from 20 cells from each field to quantify morphologic changes.

### 2.4 Prolactin measurement

The concentration of PRL was measured by the electrogenerated chemiluminescence immunoassay (ECLIA) method using ECLusys Prolactin III reagent (Roche Diagnostics, Basel, Switzerland) and Cobas 6000 (Roche Diagnostics). HESC culture media was collected on day three of decidualization for PRL measurement.

### 2.5 Total RNA extraction and quantitative real‐time reverse transcriptase PCR

Total RNA extraction from HESCs was performed using miRNeasy Mini Kit (Qiagen, Hilden, Germany). The reverse transcription for synthesis of cDNA from extracted total RNA was performed using BioScript reverse transcriptase (Bioline, London, UK). *PRL*, *IGFBP1*, forkhead box O1 (*FOXO1*), and *WNT4* mRNA expression were analyzed by real‐time reverse transcriptase PCR (RT‐qPCR). RT‐qPCR was performed with PowerUP SYBR Green PCR Master Mix (Thermo Fisher Scientific) and the PikoReal 96 Real‐Time PCR system (Thermo Fisher Scientific). Primer sequences for each gene are shown in Table 1. The mRNA expression levels relative to those of *GAPDH* were calculated by the 2^−ΔΔ^ *^Ct^* method.^16^

**TABLE 1:** Primer sequence used in real‐time RT‐PCR

Glyceraldehyde phosphate dehydrogenase (*GAPDH)*	Forward: 5′‐CGACCACTTTGTCAAGCTCA‐3′ Reverse: 5′‐AGGGGTCTACATGGCAACTG‐3′
Insulin‐like growth factor‐binding protein 1 (*IGFBP1)*	Forward: 5′‐CTGCGTGCAGGAGTCTGA‐3′ Reverse: 5′‐CCCAAAGGATGGAATGATCC‐3′
Prolactin (*PRL)*	Forward: 5′‐CTACATCCATAACCTC TCCTCA‐3′ Reverse: 5′‐GGGCTTGCTCCTTGTCTTC‐3′
Wnt family member 4 (*WNT4)*	Forward: 5′‐CAT GCAACAAGACGTCCAAG‐3′ Reverse: 5′‐AAGCAGCACCAGTGGAATTT‐3′
Forkhead box O1 *(FOXO1)*	Forward: 5′‐ATTCGGAATGACCTCATGGA‐3′ Reverse: 5′‐TTTTAAGTGTAACCTGCTCACTAACC‐3′

### 2.6 Actin staining analysis

Cells were fixed with 4% paraformaldehyde in phosphate‐buffered saline (PBS; Nacalai Tesque, Inc, Kyoto, Japan) for 10 minutes, washed in PBS, and permeabilized with 0.5% Triton X‐100 at room temperature. Actin was stained with the fluorescent marker Acti‐stain™ 555 phalloidin (Cytoskeleton, Inc, Denver, CO, USA). Cells were mounted with mounting media containing 4,6‐diamidino‐2‐phenylindole (DAPI; Vector Laboratories, Inc, Burlingame, CA, USA) and visualized using a fluorescent laser microscope (Axiocam MRm; Carl Zeiss AG, Oberkochen, Germany).

### 2.7 Statistical analysis

PRL or RT‐qPCR analysis was conducted on six sets of measurements. Statistical analyses were performed by two‐tailed Student's *t* test for comparisons within two groups. All graphs with error ranges denote mean ± standard error of the mean (SEM). A *P*‐value of <.05 was considered significant.

## 3 RESULTS

### 3.1 Cyclic stretch inhibits morphological change and expression of decidual markers on decidualized HESCs

**FIGURE 1:**
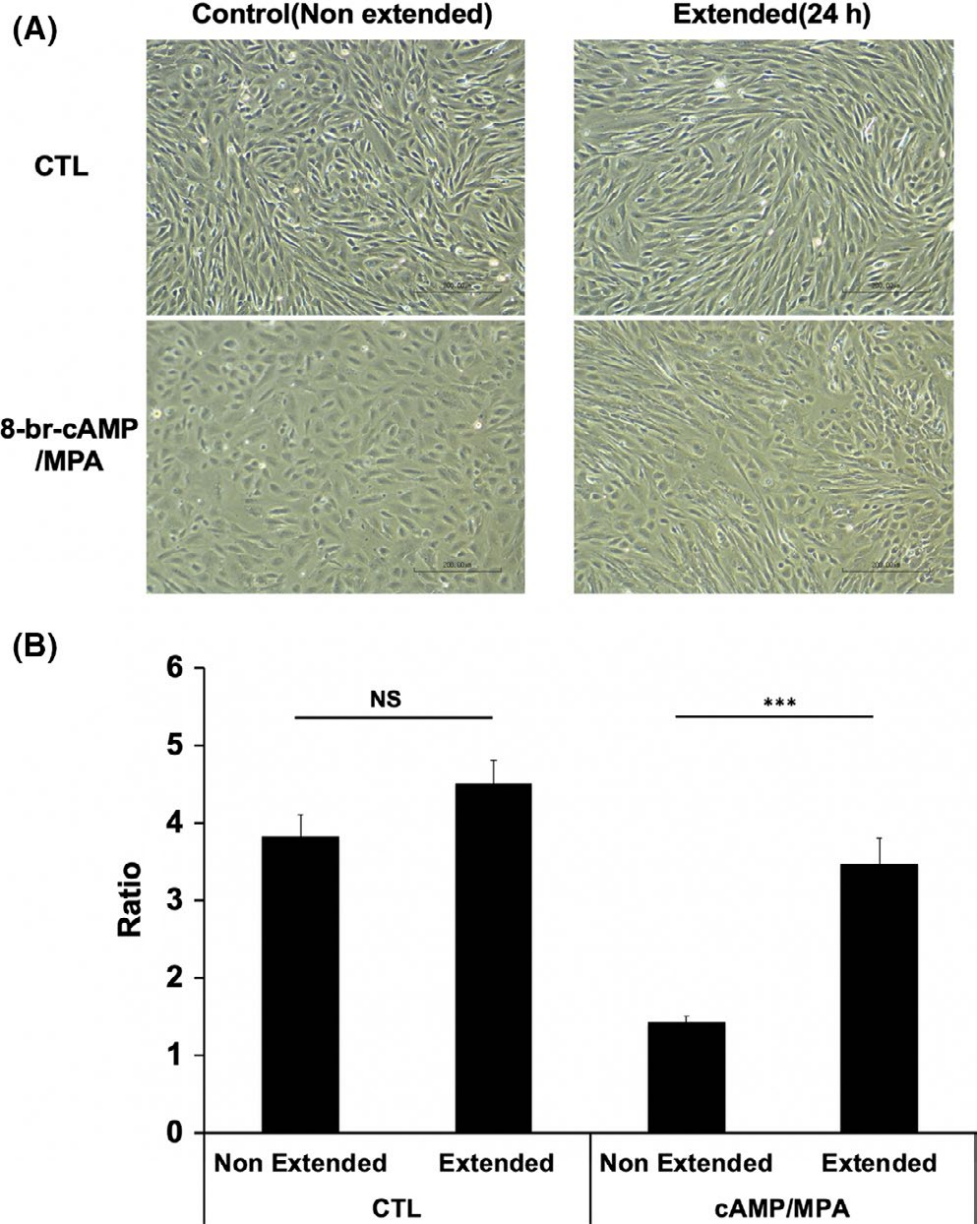
The effect of cyclic stretch on the phenotype of HESCs. HESCs were treated with or without 8‐bromo‐cAMP and 10^−6^ M MPA as decidual stimulation for three days. Decidualized and non‐decidualized HESCs were then cultured with or without cyclic stretch for 24 h. Scale bar indicates 200 μm (A). The aspect ratio of cell major axes (lengths) over minor axes (widths), with an aspect ratio of 1.0 yielding a circle, was determined for the HESC culture system by using ImageJ software. The shape indexes were calculated from 20 cells from each field of cells to quantify morphologic changes. Data shown are mean ± SEM of five biological repeats. ***P < .001 (B)

**FIGURE 2:**
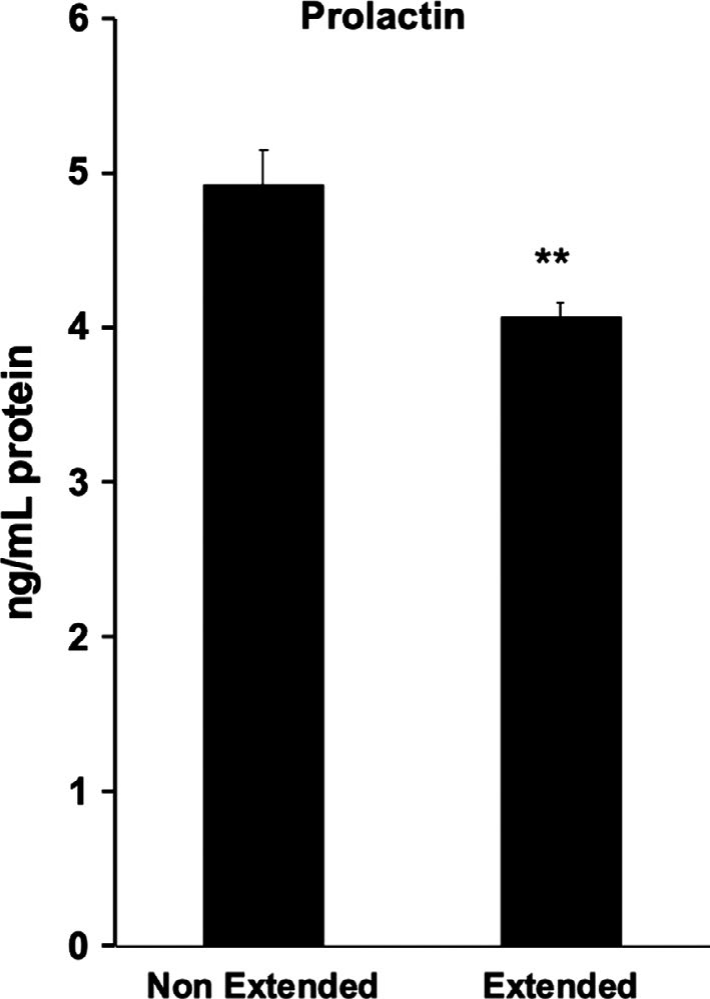
The effect of cyclic stretch on the secretion for PRL of decidualized HESCs. HESCs were treated with or without 8‐bromo‐cAMP and 10^−6^ M MPA as decidual stimulation for three days. Then decidualized and non‐decidualized HESCs were cultured with or without cyclic stretch for 24 h. These data represent the mean of PRL protein concentration in the supernatant, normalized by the total protein content. Data are shown as mean ± SEM of five individuals. **P < .01

In the absence of hormonal treatment with or without cyclic stretch, confluent primary HESCs have a spindle‐shaped fibroblast‐like appearance via light microscopy. In agreement with our previous study,^8^ treatment of decidualizing HESCs with 8‐br‐cAMP and MPA without cyclic stretch resulted in typical morphology of decidual cells characterized by the presence of larger and rounder cells with larger nuclei and abundant cytoplasm. By contrast, cyclic stretch of decidualized HESCs failed to show typical decidual transformation and instead showed an elongated shape (Figure 1A). The aspect ratio of the major axis (length) over the minor axis (width) of each cell was used to quantify the changes in cell morphology upon decidualization in the presence or absence of cyclic stretch. For non‐decidualized HESCs, cyclic stretch had no effect on the aspect ratio. By contrast, the aspect ratio of decidualized HESCs with cyclic stretch was significantly higher, indicating the relative abundance of spindle‐shaped cells compared with decidualized HESCs without cyclic stretch (Figure 1B). Furthermore, PRL secretion of decidualized HESCs with cyclic stretch was significantly reduced in comparison with decidualized HESCs kept stationary (Figure 2).

**FIGURE 3:**
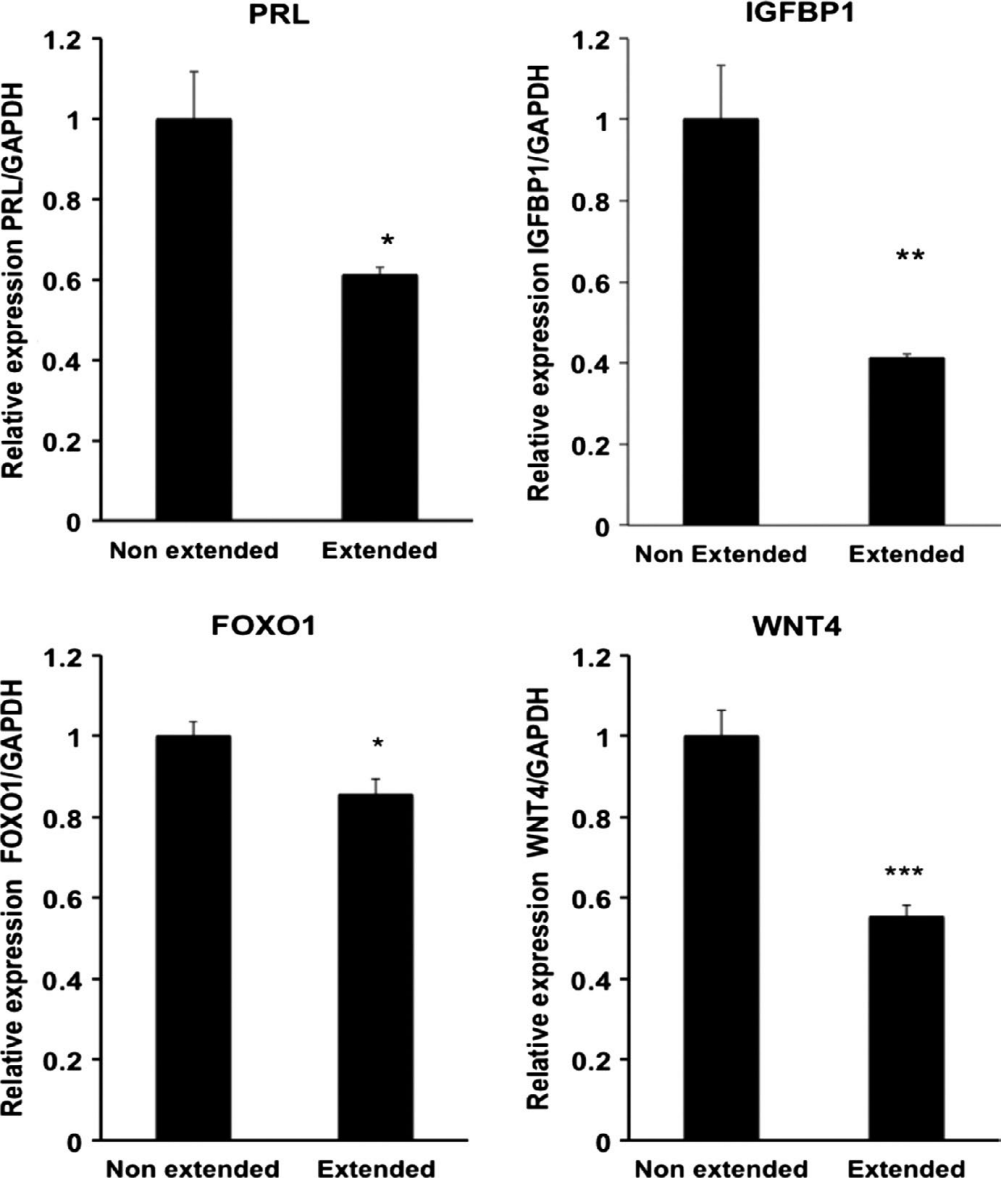
The effect of cyclic stretch on the expression of *PRL*, *IGFBP1, FOXO1,* and *WNT4* in decidualized HESCs. HESCs were treated with or without 8‐bromo‐cAMP and 10^−6^ M MPA as decidual stimulation for three days. Decidualized HESCs were then cultured with or without cyclic stretch for 24 h. RT‐qPCR analysis of *PRL, IGFBP1*, *FOXO1,* and *WNT4* transcript levels was performed in decidualized HESCs with or without cyclic stretch. Expression levels were normalized to *GAPDH*. Data are the mean ± SEM of three independent experiments **P < .01, *P < .05

Next, we analyzed the effect of cyclic stretch on the expression of major decidual makers on decidualized HESCs. Cyclic stretch significantly repressed expression of *PRL, IGFBP1, FOXO1,* and *WNT4* on decidualized HESCs as compared with decidualized HESCs kept stationary (Figure 3).

### 3.2 Alteration of F‐actin localization and cytoskeleton of decidualized of HESCs with cyclic stretch

**FIGURE 4:**
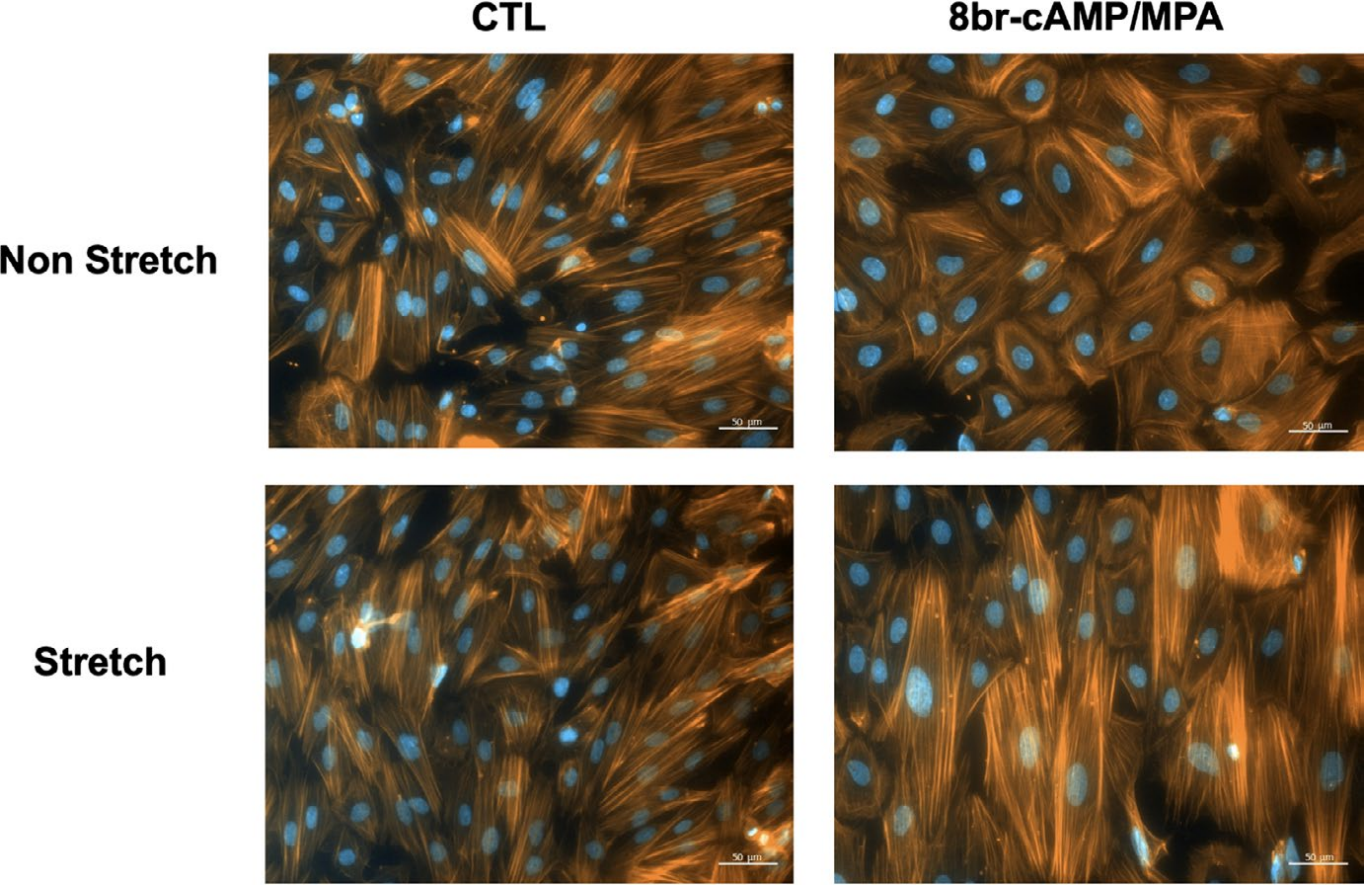
Alterations to F‐actin localization in non‐decidualized HESCs and decidualized HESCs with or without cyclic stretch for 24 h. Actin filaments were visualized with Acti‐stain™ 555 phalloidin (Cytoskeleton). Scale bar, 50 µm

A previous study reported that the actin‐based cytoskeleton contributes to both functional and morphological endometrial decidualization.^17^ Therefore, we investigated the subcellular distribution of actin stress fibers in non‐decidualized and decidualized HESCs treated with or without to cyclic stretch using Acti‐stain™ 555 phalloidin. Well‐stretched F‐actin was distributed throughout the cytoplasm in undifferentiated HESCs during both stationary and cyclic stretch conditions. In response to decidual stimulation, F‐actin was located in the periphery of decidualized HESCs kept stationary. However, we were unable to observe an alteration in F‐actin localization in decidualized HESCs with cyclic stretch (Figure 4).

## 4 DISCUSSION

The findings of the present study demonstrate that cyclic stretch hinders the morphological alteration of HESCs upon decidualization. Cyclic stretch repressed expression of decidual markers in decidualized HESCs including *PRL*, *IGFBP1*, *FOXO1*, and *WNT4*. In addition, cyclic stretch inhibited the change in F‐actin localization in decidualized HESCs. These observations suggest that cyclic stretch inhibits morphological change through biological decidual process. Therefore, abnormal uterine peristalsis may affect the implantation of the embryo.

Decidualization represents the transformation of endometrial stromal cells into specialized secretory epithelioid cells. This differentiation process is initiated in the mid‐to‐late secretory phase of the menstrual cycle and coincides with vascular remodeling and influx of specialized immune cells, mainly uterine natural killer cells and macrophages, into the stroma. The decidual process is essential for formation of a functional feto‐maternal interface as it controls endovascular trophoblast invasion, ensures tissue homeostasis, and confers resistance to oxidative stress signals.^1^ Impairment of the decidualizing process is increasingly linked to a variety of pregnancy disorders including infertility, recurrent implantation failure, recurrent miscarriages, preeclampsia, intrauterine growth restriction, endometriosis, and endometrial malignancy.^5^, ^6^, ^7^, ^8^

It has been reported that subendometrial myometrium exerts wave‐like activity (uterine peristalsis) throughout the menstrual cycle,^9^ and uterine peristalsis is markedly reduced during the implantation phase. Previous observations suggest that abnormal uterine peristalsis during the implantation period influences endometrial functions and contributes to infertility.^10^, ^11^ The present study demonstrated that cyclic stretch significantly repressed expression of decidual markers including *IGFBP1*, *PRL*, *FOXO1*, and *WNT4* on decidualized HESCs. These alterations may be through the reduction of FOXO1 gene since it has been reported FOXO1 target genes are involved PRL, IGFBP1, and WNT4.^4^ In addition, cyclic stretch of decidualized HESCs impaired decidual morphological phenotype to show a more elongated shape. It has been well‐known that decidual change of HESCs is associated with the process of mesenchymal epithelial transition (MET). In this decidual process, HESCs accumulate glycogen and lipid intracellularly, expand the rough endoplasmic reticulum, and reconstruct cytoskeletal actin filaments.^3^ In addition, we previously demonstrated that decidualized stimulation down‐regulated N‐cadherin expression and upregulated E‐cadherin expression in HESCs (cadherin switch).^18^ It is possible that high stretch cycling may inhibit this cadherin switch. However, further studies need to clarify this mechanism of impairment for decidualization. The alteration of F‐actin localization in decidualized HESCs was lost in response to cyclic stretch. These observations together suggest that cyclic stretch inhibits the morphological and functional process of decidualization. Therefore, these findings raise the possibility that uterine abnormal contraction during the implantation period leads to an impaired endometrial decidualization process and thereby contributes to the development of infertility. However, a previous report showed that cyclic stretch upregulated IGFBP1 secretion from decidualized HESCs.^12^ One possible explanation for this discrepancy could lie in different cyclic stretch conditions (a cyclic fashion at a rate of 2 vs 6 cycle/min). It has been reported that uterine movements undergo cycling at a rate of approximately 2 cycles/min in the luteal phase.^9^ Therefore, we chose this condition as abnormal uterine contraction. Several studies demonstrated different stretch conditions including the frequency of the cycle resulted in the opposite effect on the same cells.^19^, ^20^ For instance, Kubo et al reported that cell proliferation in cultured tenocytes stretched in physiologically frequent condition was augmented. Conversely, those in unphysiologically frequent condition show reduced cell proliferation.^20^ Thus, we assume it is reasonable to demonstrate opposite effect on the decidualized cells with the non‐physiological conditions compared to those with physiological conditions in Harada's study.^12^

The cytoskeleton plays a critical role in various cellular processes including migration.^21^ The organization and plasticity of the cytoskeleton are characterized primarily by forces generated by actin‐myosin interactions.^17^ Previous studies demonstrated that F‐actin was localized in the periphery of decidualized HESCs. By contrast, well‐stretched F‐actin was distributed throughout the cytoplasm in non‐decidualized HESCs.^21^, ^22^ These observations are consistent with our present microscopic findings. Furthermore, the present study demonstrated that cyclic stretch reversed the distribution of F‐action in decidualized HESCs. Interestingly, it has been reported that in women with endometriosis, the endometrium has reduced decidualization capacity compared with that of women without endometriosis.^23^ Therefore, it is possible that abnormal uterine contractions may actively contribute to sustaining the mesenchymal phenotype, retaining cell motility of HESCs against decidualization stimulation, and establishing endometriosis lesions.

In conclusion, the present study demonstrates that cyclic stretch inhibits the morphological and functional decidual process of HESCs. Our findings suggest that uterine abnormal contractions during the implantation period of fertilization impair endometrial decidualization and contribute to infertility.

## DISCLOSURE

*Conflict of interest*: The authors declare no conflict of interest. *Human rights statement and informed consent*: All procedures followed were in accordance with the ethical standards of the responsible committee on human experimentation (institution and national) and with the Helsinki Declaration of 1964 and its later amendments. Informed consent was obtained from all patients included in the study. The Institutional Review Board of the Saitama Medical University Hospital approved this study.
